# Video-assisted thoracoscopic surgery lobectomy and en bloc resection of the chest wall with incision of the costovertebral joints for non-small cell lung cancer

**DOI:** 10.1093/jscr/rjab190

**Published:** 2021-05-18

**Authors:** Shinichi Yamamoto, Masaya Sogabe, Shunsuke Endo

**Affiliations:** Department of General Thoracic Surgery, Jichi Medical University, 3311-1 Yakushiji, Shimotsuke, Tochigi 329-0498, Japan; Department of General Thoracic Surgery, Jichi Medical University, 3311-1 Yakushiji, Shimotsuke, Tochigi 329-0498, Japan; Department of General Thoracic Surgery, Jichi Medical University, 3311-1 Yakushiji, Shimotsuke, Tochigi 329-0498, Japan

## Abstract

We describe a case of lung lobectomy and resection of the rib neck and head in a lung cancer patient with an invasion of the chest wall. The tumor was located in the upper lobe, adjacent to the neck of the third rib. We performed a right upper lobectomy and en bloc resection of the third rib, including the rib neck and head, by video-assisted thoracoscopic surgery with an additional 6 cm posterior incision along the right paravertebral line. The costovertebral joint incision procedure is a useful technique to ensure tumor-free margins in cases where the tumor is located close to the rib’s neck and head.

## INTRODUCTION

Video-assisted thoracoscopic surgery (VATS) is the gold standard treatment for early-stage non-small cell lung cancer (NSCLC). In addition to being economical, VATS results in a shorter hospital stay and good quality of life post-surgery. It has recently been used in advanced cancer cases with larger lesions and invasion of the chest wall. However, the utility of VATS in advanced cases remains unknown.

Here, we describe a case of advanced lung cancer with suspected invasion of the rib neck and head wherein lobectomy and en bloc resection of the chest wall was performed by VATS.

## CASE REPORT

**Figure 1:**
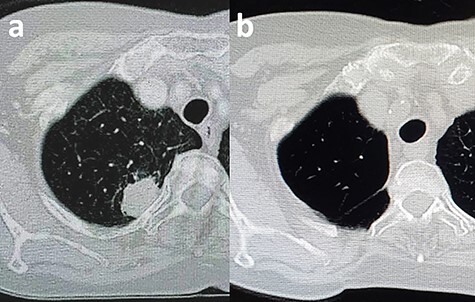
CT scan of the chest. (**a**) Shown is a tumor located adjacent to the rib’s neck and head. (**b**) This postoperation image shows the complete resection of the rib’s neck and head.

A 67-year-old woman was referred to our hospital because of cough and sputum production. A chest computed tomography (CT) scan showed a 26 × 20 mm mass in the posterior segment of the right upper lobe adjacent to the third rib’s neck and head (Fig. 1a). No mediastinal lymphadenopathy was observed. Pathological examination with transbronchial tumor biopsy revealed lung adenocarcinoma. Positron emission tomography/CT scans and brain magnetic resonance imaging indicated no evidence of distant metastasis.

**Figure 2:**
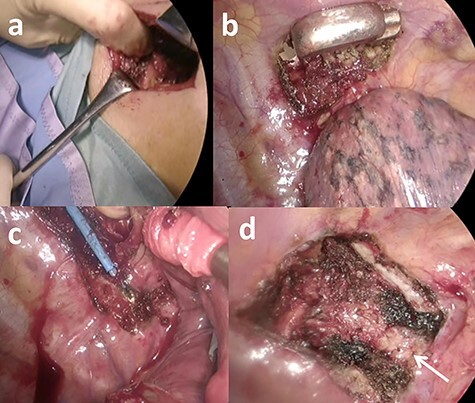
Images during the operation. (**a**) An additional posterior incision was made along the right paravertebral line. (**b**) The dorsal side of the third rib from the tumor was cut with a conventional rib cutter. (**c**) The costotransverse ligament, which holds the costovertebral joint, was divided with a conventional electrocautery. (**d**) The transverse process (arrow) was confirmed after complete resection of the rib neck and head.

Five-port VATS, which is our standard treatment, was chosen at the beginning of the operation. The five ports were ~1 cm in length. Resection of the chest wall was required to achieve adequate margins before the right upper lobectomy because the tumor had invaded the parietal pleura adjacent to the third rib. An additional 6 cm posterior incision along the right paravertebral line was performed to resect the third rib (Fig. 2a). The trapezius, rhomboid and erector spinae muscles were split upon exposure of the third rib’s neck surface. The parietal pleura of the cranial, caudal and medial sides of the tumor were excised from the thoracic cavity using electrocautery. The dorsal side of the third rib was cut 2 cm from the edge of the tumor with a conventional rib cutter (Fig. 2b). The cranial, caudal and medial sides of the intercostal muscles, intercostal vessels and costovertebral joints were separated from the additional incision using the HARMONIC ACE shears (Ethicon, Cincinnati, OH) and conventional electrocautery. The rib’s head and neck were completely separated by the transverse process by cutting the costotransverse and radiating costovertebral ligaments, which connect the costovertebral joints (Fig. 2c). The transverse process was confirmed after complete resection of the rib’s neck and head (Fig. 2d). The resected rib was attached to the upper lobe (Supplementary material), and a right upper lobectomy with systematic lymph node dissection was performed using a five-port VATS. The right upper lobe and the attached chest wall were removed the thoracic cavity using a retrieval bag. Chest wall reconstruction was not attempted because there was no evidence of a frail chest. The operation time and blood loss were 204 min and 50 mg, respectively.

There were no adverse events after the operation, and the patient was discharged on postoperative Day 7. The final pathological report revealed a 23 × 20 mm mass infiltrating the resected chest wall’s soft tissue close to the resected rib neck, and the surgical margin was negative. There was no evidence of recurrence on the 12-month follow-up CT scan (Fig. 1b).

## DISCUSSION

VATS lobectomy is the gold standard treatment for NSCLC. However, resection using VATS is often selected for advanced lung cancer due to fewer overall complications, reduced mortality rates, improved quality of life and shorter hospital stays, making it more economical [1, 2]. Good outcomes have been reported that with VATS lobectomy and en bloc resection of the chest wall with no postoperative adverse events [1, 3–5] and an expected 5-year survival of 50% [2].

In lung cancer cases with chest wall invasion, it is necessary to secure an adequate surgical margin. In patients where the tumor is close to the rib neck and head, incision of the costotransverse ligament and radiate costovertebral ligament of the costovertebral joint is very effective and can be performed without complicated devices or techniques [6, 7].

A limitation of our case is that we adopted the multiport VATS procedure. The advantage of multiple assisted ports is that it provides accurate guidance because of multiple viewpoints and appropriate lung retraction. However, reduced port surgery is minimally invasive and may be an option for thoracoscopic surgery in cases requiring rib neck and head resection if an effective retraction procedure is developed.

In conclusion, VATS lobectomy plus resection of the chest wall with the costovertebral joint incision is a very effective procedure in cases where the tumor is close to the rib’s neck and head.

## CONFLICT OF INTEREST STATEMENT

None declared.

## FUNDING

None.

## Supplementary Material

VATSCWR_rjab190
